# *Suppressor of Fused* Is Required for Determining Digit Number and Identity via *Gli3/Fgfs/Gremlin*

**DOI:** 10.1371/journal.pone.0128006

**Published:** 2015-05-22

**Authors:** Jianying Li, Qihui Wang, Ying Cui, Xueqin Yang, Yan Li, Xiaoyun Zhang, Mengsheng Qiu, Ze Zhang, Zunyi Zhang

**Affiliations:** 1 Zhejiang Key Laboratory for Organogenesis and Regenerative technology, Institute of Developmental and Regenerative Biology, College of Life and Environmental Sciences, Hangzhou Normal University, Hangzhou 310036, China; 2 Department of Anatomical Sciences and Neurobiology, University of Louisville, Louisville, Kentucky 40292, United States of America; 3 Department of Ophthalmology, Tulane University Medical Center, New Orleans, Louisiana 70112, United States of America; Instituto Gulbenkian de Ciência, PORTUGAL

## Abstract

The anterior-posterior patterning of the vertebrate limb bud requires closely coordinated signaling interactions, including Sonic Hedgehog (Shh)-mediated counteraction of the Gli3 transcription factor in the distal and posterior mesenchyme of the limb bud. Suppressor of Fused (Sufu), an intracellular negative regulator of Shh signaling via Gli2 and Gli3, is implicated in early development of the mouse limb bud. However, how Sufu is involved in the genetic regulation of limb bud patterning still remains elusive. In this study, we show that the conditional deletion of Sufu in the mesenchyme of the early limb bud results in polydactyly with loss of digit identity and supernumerary bones in the wrist and the ankle. These pattern alterations are associated with anterior expansion of HoxD genes located at the 5’ end of the cluster. By focusing on gene expression analysis of *Shh/Gremlin1/Fgf* signaling critical for the establishment and maintenance of anterior-posterior patterning, we show that early response to loss of Sufu involves anterior prolongation of *Fgf4* and *Fgf8* expression in the apical ectodermal ridge at E10.5. We also reveal the anterior activation of Shh-dependent posterior markers *Ptc1*, *Gli1* and *Gremlin* in limb buds lacking Sufu. Furthermore, we find that loss of *Sufu* leads to attenuated levels of repressor Gli2 and repressor Gli3 in the early limb bud. Moreover, expression of *Hand2* is activated in the entire limb bud at the early outgrowth stage in the mutant lacking *Sufu*. Thus, we provide evidence that Sufu is involved in the genetic network that restricts the posterior expression of *Gli2/3/Hand2* and *Gremlin/Fgf* in limb bud patterning.

## Introduction

Anterior-posterior (A-P) patterning and outgrowth of the vertebrate limb bud are coordinated by two critical centers during limb bud development [1–3]: the zone of polarizing activity (ZPA) [4, 5] in the posterior mesenchyme and the apical ectodermal ridge (AER) in the distal ectoderm [6]. Sonic hedgehog (Shh), the secreted signal molecule, is expressed in the ZPA and is sufficient for the determination of polarizing activity [5]. When *Shh* is ectopically expressed in the anterior limb bud mesenchyme, digit duplications are induced, resulting in extra digits (polydactyly) [5]. Recent studies have established that two critical regulatory signaling pathways, closely coordinated between Shh and Fibroblast Growth Factors (FGFs), are critical for the determination of limb bud patterning. In these models, Gremlin, the secreted antagonist for Bone Morphogenetic Proteins (BMPs), relays Shh signaling from the posterior mesenchyme and positively regulates the expression of *Fgfs* (*Fgf4* and *Fgf8*) in the AER [7, 8]. The AER, by releasing FGFs, is required for underlying mesenchymal proliferation and survival [8]. FGFs in turn maintain the expression of *Shh* in the posterior mesenchyme [2, 3]. They act as transcription feedback loops in the transcriptional regulation of each other [1–3, 7].

Shh signaling in the vertebrate is mediated by Gli transcription factors (Gli1-3) [9], Glis in turn induce the expression of Shh targets including *Gli1* and *Ptc1* [9, 10]. Among these Glis, Gli3 is the only one essential for patterning of the limb bud, while the targeted deletion for Gli1 or Gli2 displays normal limb development comparable to the wild type [9, 11–13]. Mutations of GLI3 in humans are associated with malformation of limb development [14, 15]. Distinct genetic deficiencies of Gli3 allele in mice exhibit the polydactylous phenotypes [16–18]. Notably, homozygous *Gli3* ^-/-^ displays more severe pre-axial polydactyly than heterozygous *Gli3* ^+/-^ [19], indicating that Gli3 functions as a regulator in the identity of digit patterning along the posterior to anterior axis.

It has been shown that genetic antagonism of Gli3 is required for the restriction of posterior mesenchymal expression of transcription factors *Hand2* and 5’ end HoxD clusters [11, 20], leading to the pre-patterning the posterior-anterior mesenchyme prior to the induction of *Shh* [2, 11, 20]. The absence of Shh signaling promotes formation of the Gli3 repressor (Gli3R) by proteolytic cleavage of the full-length Gli3 (Gli3F) [21, 22]. The level of Gli3R was inhibited by premature activation of Shh signaling that caused the AER’s and the ZPA's failure to form [23]. Thus, in the early pre-patterning limb bud, Gli3R represents the majority of Gli3 in the anterior mesenchyme [22, 23].

In mammals, Suppressor of Fused (Sufu), an intracellular PEST-domain- containing protein, is the negative regulator of Gli activities, both in the presence and absence of cilia [24–26]. Biochemical analyses have established that in the absence of Shh signal, Sufu restrains Gli3 in the cytoplasm, promoting its processing into a repressor. Binding of hedgehog ligands triggers the dissociation of Sufu from Gli3, thus preventing the formation of Gli3R and allows Gli3 to move into the nucleus as an activator [26–28]. Targeted ablation of mouse *Sufu* results in global activation of hedgehog signaling and early embryonic lethality, indicating that Sufu is critical for mouse development [29, 30]. Inactivation of *Sufu* has revealed its critical roles in embryonic patterning, including ventralization of the neural tube [29, 30] and impaired mid-hindbrain patterning [31]. It is noted that *Sufu* is mostly expressed in developing embryonic structures where the patterning is under control by Shh signaling, including in the developing limb bud [32, 33]. However, how Sufu is genetically involved in the anterior-posterior limb bud patterning surprisingly remains elusive.

In the current study, we approach the genetic roles of Sufu in regulating early limb bud patterning by using *Dermo1-Cre* mediated tissue specific deletion of Sufu in the limb bud mesenchyme. We show that the ablation of *Sufu* leads to the polydactylous digit defect, indicating the functional requirement of Sufu for determination of digit number and identity. We further demonstrate that the Shh signaling*/Gremlin/Fgfs* are ectopically expanded to the anterior limb bud in the *Sufu* mutant. Notably, the levels of Gli2R and Gli3R are reduced in the *Sufu* mutant, coincident with the ectopic expression of *Hand2*. Our study suggests that Sufu is integrated into the gene network that restricts the activation and maintenance of the Shh pathway to the posterior limb bud mesenchyme.

## Materials and Methods

### Ethics Statement

All animal experiments were carried out in strict accordance with the Guide for the Care and Use of Laboratory Animals at Hangzhou Normal University, and were approved by the Committee on the Ethics of Animal Experiments of (HNLA-2012-125). Mice were routinely maintained in a specific pathogen free (SPF) animal facility and received access to water and chow *ad libidum*. Enough space was provided for mice that no more than 5 animals were kept in a small cage and 10 animals in a large cage. The mice were sacrificed by cervical dislocation for sample collection. All efforts were made to minimize the usage quantity of the mice. There was no in vivo animal experiment in our study.

### Mice

Exon 7 of the mouse *Sufu* gene was flanked by two *LoxP* sites to generate a conditional *Sufu* targeting mouse strain (S1 Fig). Homozygote (*Sufu* ^*fx/fx*^ *)* mice are maintained on C57BL/6/129 background. *Dermo1-Cre* and *EIIa-Cre* mice and R26R mice were purchased from the Jackson Laboratories (Maine), and maintained on a C57BL/6 background. The morning when vaginal plugs were detected was considered as embryonic day zero. Totally, about 20 male and 320 female mice were used for sample collection.

### Skeletal preparation and digit analysis

Skeletal preparations with Alcian blue and Alizarin red staining were performed as previously described [34]. Briefly, skinned mice were fixed in 100% ethanol for 2 days, dehydrated in 100% ethanol and acetone for 2 days. Samples were stained in mixture solution composing of 1 volume (vol) of 0.1% Alcian Blue, 1 vol 0.1% Alizarin Red solution, 1 vol acetic acid, 17 vol ethanol. Samples were then treated by KOH and cleared by glycerol.

### Criteria for determination digit number and limb pattern

The criteria as described by Joyner and colleagues [18] were modified and used for the determination of digit number and autopod patterning in this study. According to these criteria, the term “digit” in this manuscript means a complete distal-proximal digit ray, composed of a metacarpal/metatarsal bone in the proximal side and associated phalanges on the distal side. Cartilaginous fragments forming in the metatarsal position without ossification are not termed as digits.

### X-gal staining and *in situ* hybridization

To detect the Cre activity in embryonic limb buds, embryos carrying *R26R/Dermo1-Cre* alleles were collected and fixed for whole mount and cryostat section X-Gal staining as previously described [35, 36]. For detection of gene expression pattern, embryos were fixed in 4% paraformaldehyde (PFA) overnight. Whole mount and section in situ hybridization were performed following the procedures as previously described [34, 35].

### Immunohistochemistry and western blot analyses

To detect the expression of Sufu in the wild type and *Sufu* mutant limb buds, the samples with horizontal paraffin sections were subjected to immunohistochemical analysis with Sufu antibody (1:200) according to the method depicted previously [35]. For immunoblot analysis, whole cell protein lysates were prepared from dissected embryonic limb buds at embryonic day 10.25 (E10.25), E10.5, and E11.5. 40 μg of protein lysates were run on a 7.5% PAGE gel and transferred onto membrane for blot analysis as described previously [35]. Sufu antibody (1:1000) and Gli2 antibody (1:1000) were purchased from Abcam (ab52913 and ab26056, respectively). Gli3 antibody (1:1000) was from R&D System (AF3690). The gray values of immunoblot bands were evaluated with Image-Pro Plus software (version 6.0). The relative expression level of a protein was calculated by dividing its gray value by that of the corresponding reference protein. The significance of expression differentiation between wild type and mutant samples was analyzed with Student’s *t* test.

## Results and Discussion

### *Sufu* is expressed in the limb bud mesenchyme during early development

**Fig 1:**
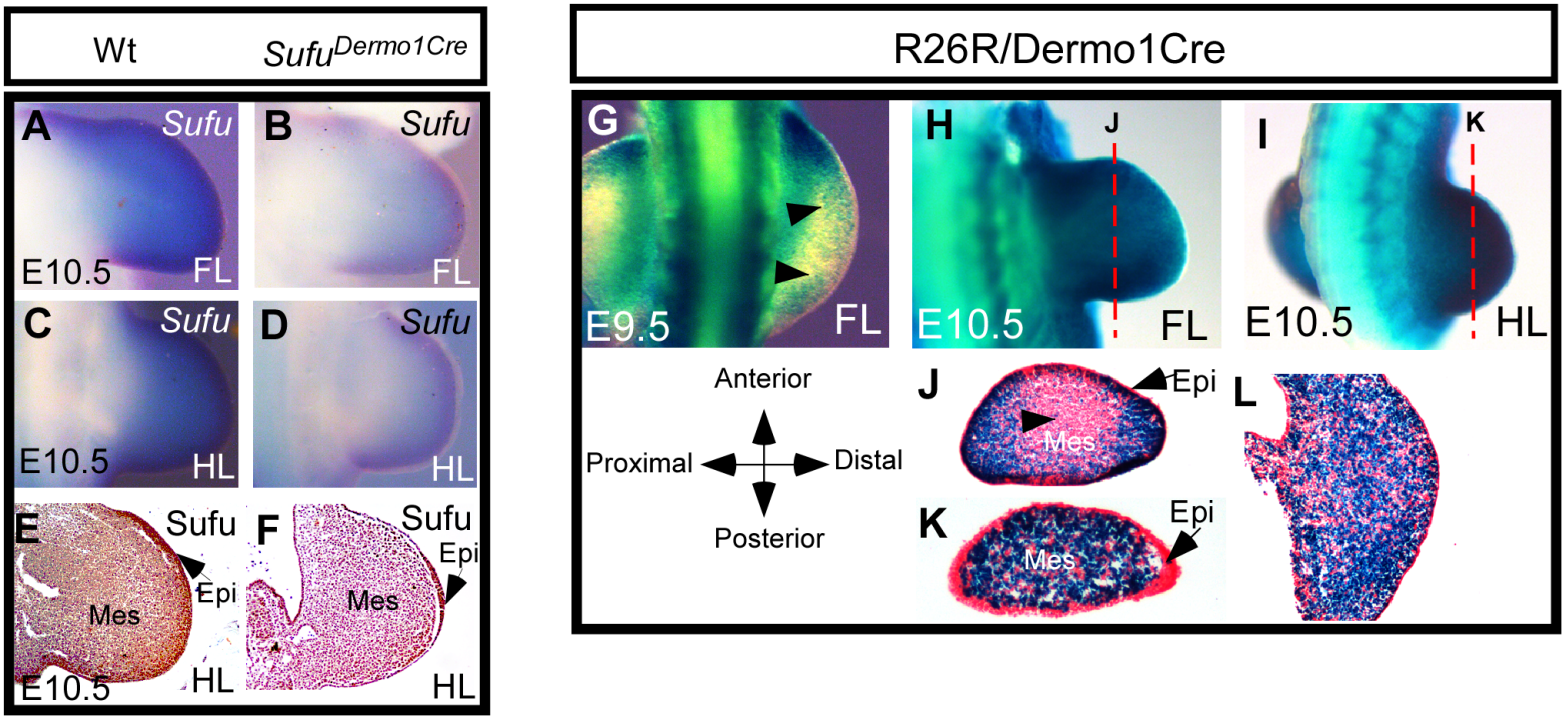
Deletion of Sufu in the mesenchyme of limb buds with *Dermo1-Cre* mouse. A-D: Whole mount in situ hybridization shows the expression of Sufu in fore- (A, B) and hind- (C, D) limb buds at E10.5. Noted the significantly reduced Sufu transcript in the *Sufu* ^*Dermo1Cre*^ mutant limb buds (B, D), in comparison with the wild type (A, C). E-F: Immunohistochemical staining of the limb bud section using antibody against Sufu protein in wild type (E) and *Sufu* ^*Dermo1Cre*^(F). G-L: *X-Gal* staining of *R26R/Dermo1-Cre*. Whole-mount samples show Cre activity in the limb buds (G-I). J and K are sections of H and I, respectively. L is a horizontal section of I. Note the weaker staining in the core mesenchyme of the forelimb bud (arrowhead in J), compared to the hind limb bud (K). No Cre activity was detected in the limb bud epithelium (arrows in J and K). Mes: mesenchyme; Epi: epithelium; FL: forelimb bud; HL: hind limb bud.

Whole mount in situ hybridization was conducted in the developing limb buds between E9.5 and E11.5 to display the *Sufu* RNA expression using mouse *Sufu* riboprobe. *Sufu* transcripts were found in limb buds throughout these stages (Fig 1A, 1C and S2 Fig), consistent with previous observations in chick and mice [32, 33] and predominantly expressed in the mesenchyme and slightly in the ectoderm (S2 Fig).

*Sufu* null mutants are lethal before limb bud outgrowth [29, 30]. To elucidate the genetic role of Sufu in limb bud development, we generated a conditional Sufu targeting mouse strain by flanking exon 7 with two *LoxP* sites (*Sufu* ^*flox*^) (S1 Fig). The *EIIa-cre* mouse line carrying a cre transgene under the control of the adenovirus EIIa promoter was used for germ line deletion of the LoxP-flanked Sufu gene [37]. Deletion of Sufu with *EIIa-Cre* mouse led to embryonic lethality at E9.5 with the same phenotype as Sufu null mutants [29, 30], indicating a loss of Sufu function (S1 Fig). Ablation of Sufu in the limb bud mesenchyme was achieved by crossing *Sufu* ^*flox*^ to mouse line carrying *Dermo1-Cre* allele to generate *Sufu* ^*fx/fx*^ */Dermo1-Cre* (*Sufu* ^*Dermo1Cre*^). The *X-Gal* staining of *Sufu* ^*fx/+*^ */R26R* reporter mice showed that *Dermo1-Cre* line exhibited weaker Cre recombination activity in the forelimb bud mesenchyme at E9.5, but this activity strengthened in majority of the mesenchyme in both fore- and hind limbs at E10.5 (Fig 1G–1L). Whole mount in situ hybridization showed that *Sufu* expression was significantly diminished in *Sufu* ^*Dermo1Cre*^ limb buds, in comparison with wild type (Fig 1A–1D). Immunohistochemical staining of *Sufu* ^*Dermo1Cre*^ mutant also confirmed that Sufu protein was indeed absent in the limb bud mesenchyme but retained in the epithelium (Fig 1E and 1F), indicating a *Dermo1-Cre* targeted removal of Sufu in the mesenchyme.

### Loss of *Sufu* in the limb bud mesenchyme results in polydactyly

**Fig 2:**
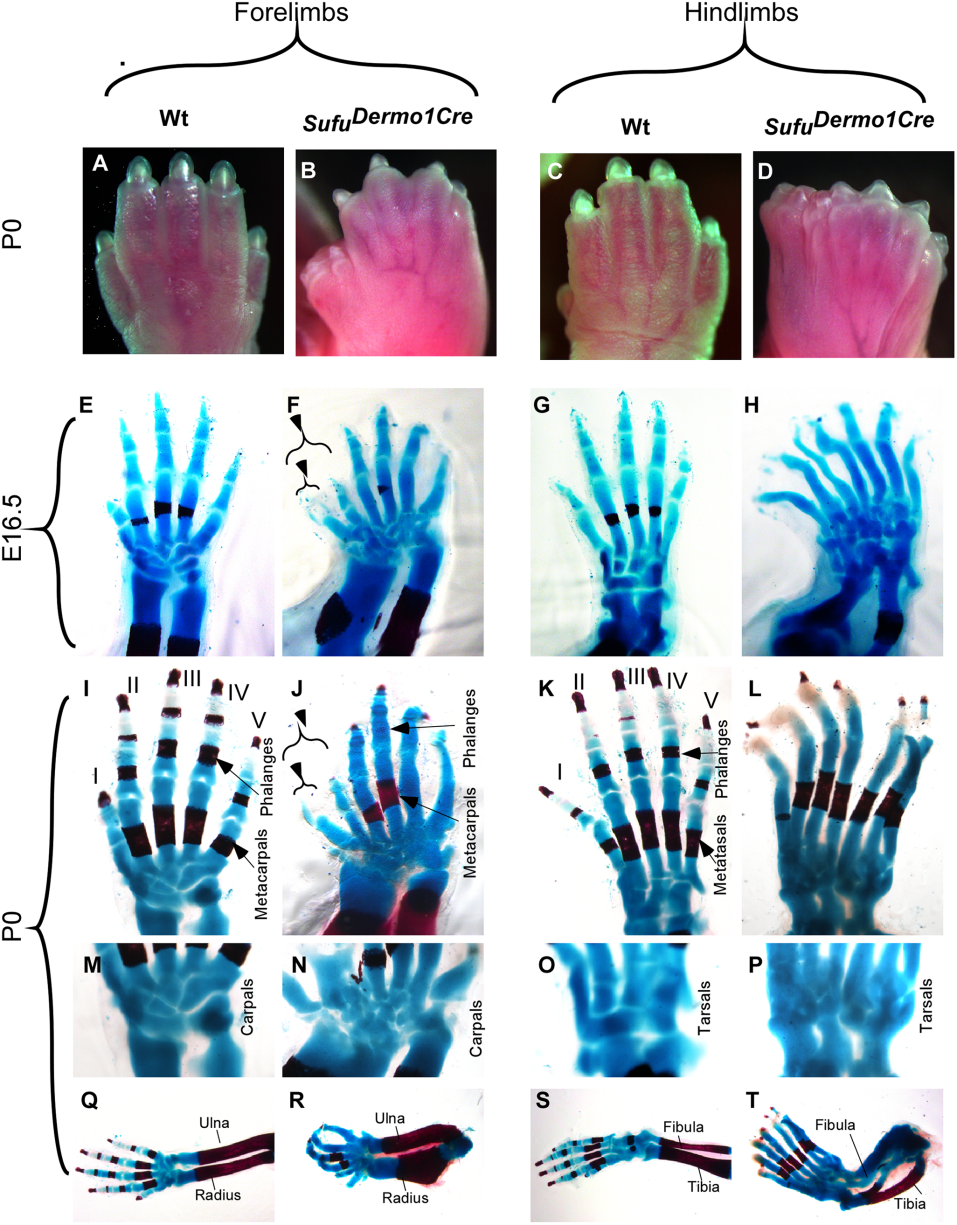
Sufu deletion results in the loss of digit identity and polydactyly. A-D: Abnormalities of autopods in *Sufu* ^*Dermo1Cre*^. E-T: Skeletal preparations of Alcian blue/Alizarin Red staining show the alteration of digit number and identity. Note the varying abnormalities in the fore- and hind limbs. Split of the anterior-most digit occurred in forelimb of the *Sufu* mutant (F, J versus E, I and arrowheads). Mutant hind limb developed 7 digits with complete loss of identity (H, L versus G, K). Phalangeal numbers distal to the metatarsals/metacarpals are not distinguishable in mutants (J, L versus I, K in wild type). Both fore- and hind limbs developed supernumerary bone elements in the wrist and the ankle of the mutant (N, P versus M, O in wild type). Note that the forelimb developed thicker radius (R versus Q), while the hind limb contained splitting of both the fibula and tibia (T versus S).

*Sufu* ^*Dermo1Cre*^ mice died shortly after birth due to the craniofacial defects (data not shown). Mutant pups developed grossly observable abnormalities on the digit pattern for both forelimbs and hind limbs, compared to wild type controls (Fig 2A–2D). To assess the defects in detail, skeletal staining with Alcian Blue for cartilage and Alizarin Red for ossified bone were performed at E16.5 and P0. The autopod skeletons of wild type mice are asymmetrically arranged such that digit one consists of two phalanges; the four posterior digits consist of three phalanges. There is proper articulation between the proximal phalange and its adjoining metapodial elements (metatarsal and metacarpal) and between each phalange (Fig 2E, 2I, 2G and 2K). *Sufu* ^*Dermo1Cre*^ mutant limbs developed polydactlylous autopods (Fig 2F, 2J, 2H and 2L). The forelimb in *Sufu* ^*Dermo1Cre*^ exhibited splitting of the anterior-most cartilaginous digit forming the distal-most bifurcations (arrowheads in Fig 2F and 2J, *n* = 10/10). Digits 2–4 are all composed of three phalanges were always distinguishable (Fig 2F and 2J, *n* = 10/10), while the posterior-most digit was always a poorly differentiated cartilaginous element without identifiable phalanges (Fig 2F and 2J, 10/10). Mutant hind limbs (9/10) contained 7–8 digits that completely lacked identity (Fig 2H and 2L), in comparison with the wild type (Fig 2G and 2K), indicating the requirement of Sufu for the determination of the digit number and anterior/posterior polarity. The polydactyly in mutant hind limb was more severe than in the mutant forelimb (Fig 2F, 2H, 2J, and 2L), which might be caused by differential Cre activity between the fore- and hind-limb buds (Fig 1J and 1K). Moreover, the articulations between the metatarsal and metacarpal bones in the mutant limb were poorly differentiated compared to that in the wild type limb (Fig 2E–2L). Skeletal preparations also revealed that the bone ossification in the phalanges was delayed (Fig 2I–2L), which was similar to that of *Gli3* deficiency [11, 18, 20].

### Sufu deletion resulted in limbs with supernumerary tarsal/carpal bones

One unexpected limb defect in *Sufu* ^*Dermo1Cre*^ was the formation of the supernumerary tarsals and carpals (Fig 2M–2P). These supernumerary bones are unidentifiable as defined bones due to incomparable shape to corresponding bones in the wild type (Fig 2M and 2O). These phenotypes were found neither in *Gli3* null mutants [11, 19], nor in the constitutive activator/repressor Gli3 mutants [16, 17]. Moreover, the radius in the mutant forelimb was overgrown (Fig 2Q and 2R), and the tibia and fibula in the mutant hind limbs seem to be split in most of the mutants (Fig 2S and 2T) (10/12). These data suggest the requirement of Sufu for patterning of both the autopod and the zeugopod, consistent with its function in mediating the chondrocyte differentiation in the long bone growth plate [38].

### Sufu deficiency disrupted the posterior restriction of 5’ HoxD gene expression

**Fig 3:**
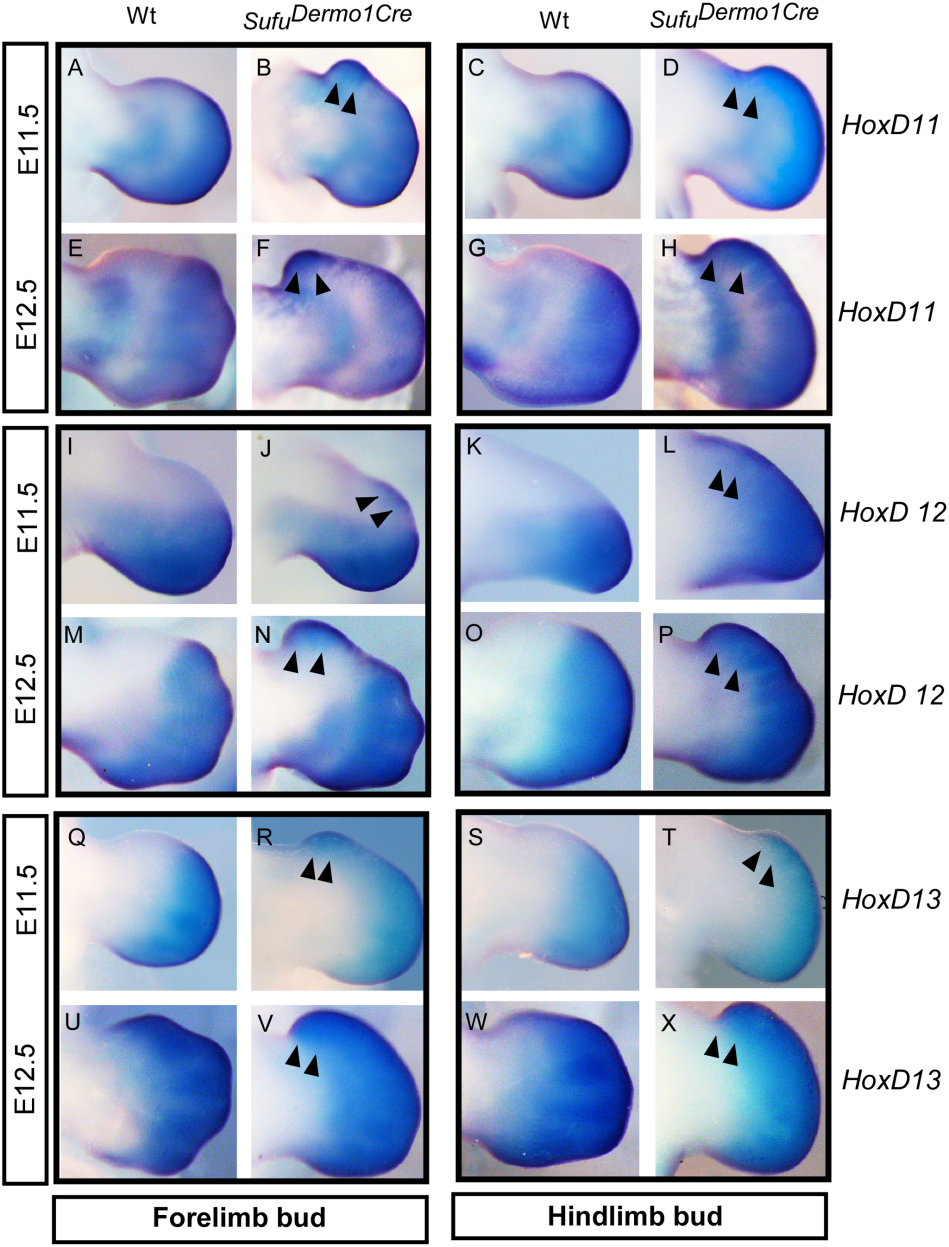
Expression of 5’ HoxD genes in *Sufu* deletion. A-X: Whole-mount in situ hybridization of limb buds showing the alteration of *HoxD11*, *HoxD12*, and *HoxD13* in *Sufu* ^*Dermo1Cre*^ at E11.5 (A-D; I-L; Q-T) and E12.5 (E-H; M-P; U-X). Note the anteriorly expanded expression for all three genes.

The posterior nested expression of 5’HoxD transcription factors were critically regulated in limb patterning. *HoxD11* is critical for zeugopod and carpal/tarsal patterning, while *HoxD12* and *HoxD13* are essential for autopod patterning [16, 39]. In mouse limb development, *HoxD11* is critical for zeuogopod and carpal/tarsal patterning. In *Sufu* ^*Dermo1Cre*^ limb buds, *HoxD11* expression was anteriorly up-regulated in both the fore- and hind-limb buds at E11.5 and E12.5 (Fig 3A–3H), suggesting a molecular alteration for excessive growth of the zeugopod. In early limb buds during E11.5 and E12.5, *HoxD12* expression in the wild type was restricted to the posterior mesenchyme (Fig 3I, 3M, 3K and 3O), corresponding to digit condensations except digit 1. *HoxD13* was expressed similarly but more anteriorly than *HoxD12* at E11.5 and in all digit condensations at E12.5 (Fig 3Q, 3U, 3S and 3W). However, transcript localizations of all three HoxD genes were anteriorly expanded in *Sufu* ^*Dermo1Cre*^ (arrowheads in Fig 3B, 3F, 3D, 3H, 3J, 3N, 3L, 3P, 3R, 3V, 3T and 3X), which suggest an altered spatial expression of 5’HoxD genes in early and late patterning of limb buds.

### Sufu deletion causes anterior expansion of AER and ZPA activity

Most polydactylous limb phenotypes, if not all, are associated with the alteration of limb bud polarity. AER and ZPA are two signaling centers that control the early limb bud polarities. To identify the genes involved in the development of polydactylous defects in *Sufu* ^*Dermo1Cre*^, we analyzed the expression of molecules that are potentially involved in AER and ZPA signaling interactions before manifestation of any morphological defects.

**Fig 4:**
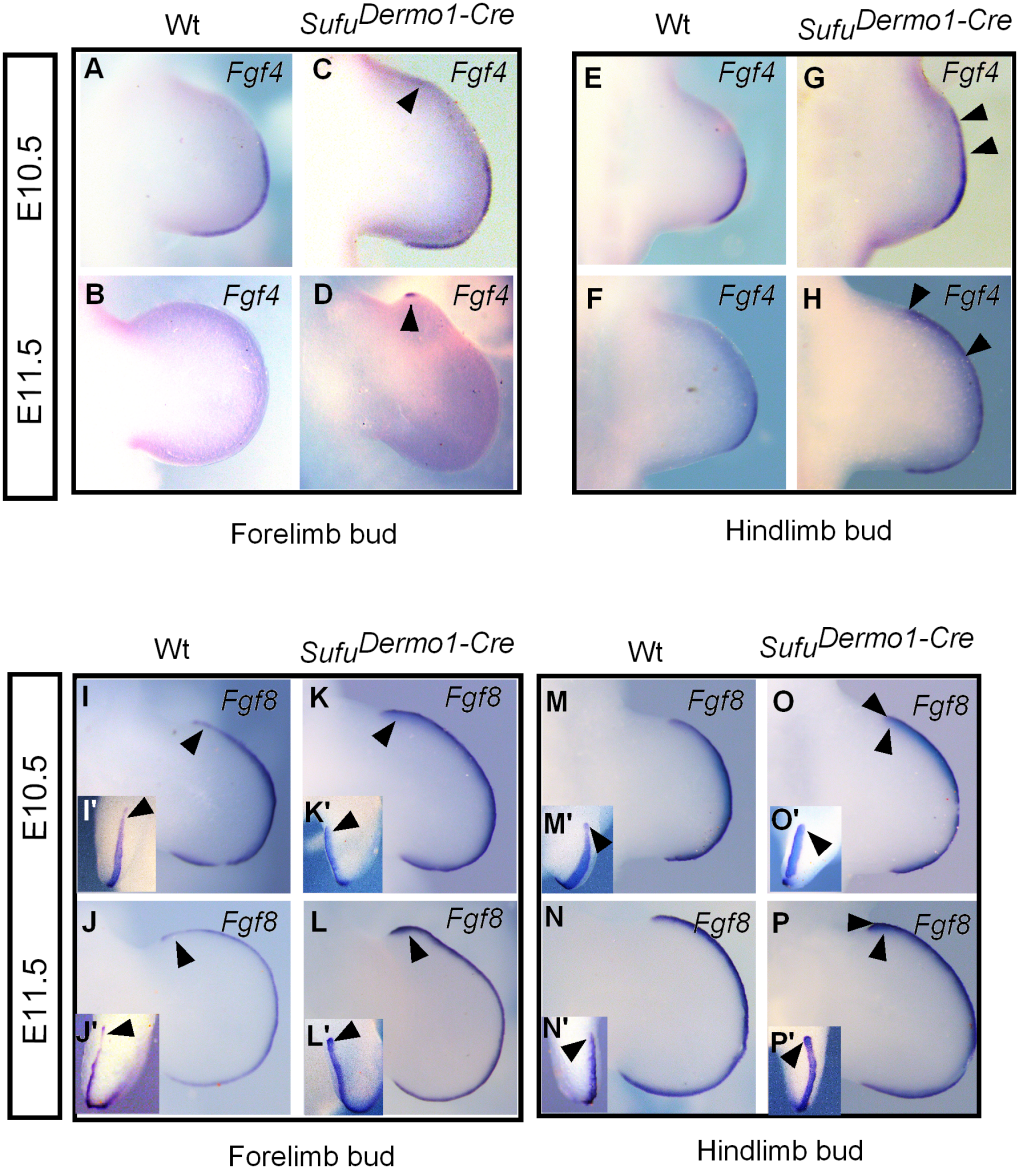
Expression of AER molecular markers relevant to the activation of the Shh/Fgf loop. A-H: Whole-mount in situ hybridization of limb buds showing the expression of *Fgf4* in the AER was anteriorly expanded in *Sufu* ^*Dermo1Cre*^. The *Fgf4* transcript in the anterior AER was detected at E10.5 (arrowheads in C, G versus A, E in the wild type), which became evident at E11. 5 (arrowheads in D, H versus B, F in the wild type). I-P: *Fgf8* expression along the AER was intensified and expanded in *Sufu* ^*Dermo1Cre*^ (K, O, L, P versus I, M, J, N in the wild type). I’-P': Front views of AER (inserts in I-P, respectively).

*Fgf4* expression within the AER was anteriorly expanded in *Sufu* ^*Dermo1Cre*^ in early patterning limb buds at E10.5 (Fig 4C and 4G) in comparison with that in the wild type (Fig 4A and 4E). At E11.5, when *Fgf4* was down-regulated in the AER of the wild type control (Fig 4B and 4F), its transcripts in *Sufu* ^*Dermo1Cre*^ was detected in the anterior ectoderm (Fig 4D and 4H). *Fgf8* was expressed in the entire AER at E10.5 in the wild type limb bud, along the anterior/posterior axis (Fig 4I, 4I’, 4M, 4M’). At E11.5, the thickness of the AER and the expression of *Fgf8* were decreased in the wild type (Fig 4J, 4J’, 4N and 4N’). However, in *Sufu* ^*Dermo1Cre*^, *Fgf8* expression was intensified in the anterior-most ectodermal ridge of fore- and hind limb buds at both stages (Fig 4K, 4K’, 4L, 4L’, 4O, 4O’, 4P and 4P’). Thus, the anterior expansion of *Fgf4* and up-regulation of *Fgf8* in the AER reflects the expanded AER in *Sufu* ^*Dermo1Cre*^.

**Fig 5:**
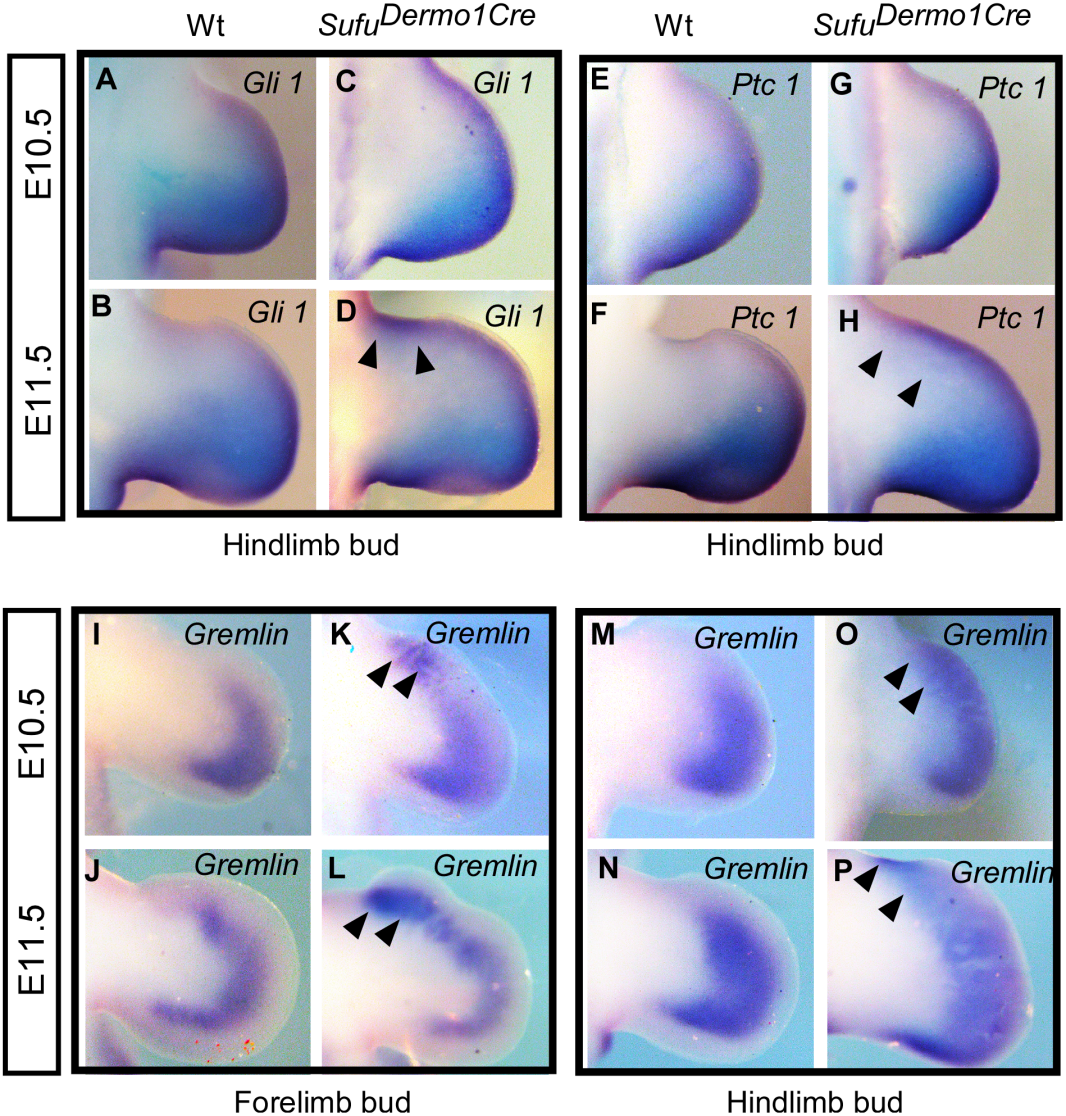
Expression of Shh regulated posterior genes. A-H: Whole mount in situ hybridization of hind limb buds showing the expression of Shh targeted genes. The expressions of *Gli1* (A, B and arrowheads in C, D) and *Ptc1* (E, F and arrowheads in G, H) were activated in the anterior limb bud mesenchyme in *Sufu* ^*Dermo1Cre*^. I-P: Whole-mount in situ hybridization of limb buds showing that *Gremlin* expression was anteriorly expanded in both the fore- (K, L and arrowheads versus I, J) and hind limb buds (O, P and arrowheads versus M, N).

Our data revealed that in contrast to the wild type forelimb bud, where the *Shh* was undetectable from E11.5 and on, in the *Sufu* mutant, *Shh* was still detectable in the posterior forelimb bud at E11.5 (S3 Fig). Interestingly, *Shh* was also detected in the anterior forelimb bud of the *Sufu* mutant at E11.5, albeit weakly, and becomes more evident at E12.5 (S3 Fig). Thus, our results indicate an ectopic activation of *Shh* expression in the absence of Sufu. There were no differences in the *Shh* expression pattern in the hind limb buds (S3 Fig). Additionally, expression of *Gli1* and *Ptc1*, two downstream target genes of Shh, were altered in limb buds lacking Sufu. The expression of *Gli1* was anteriorly activated by E11.5 (Fig 5C and 5D versus 5A and 5B in wild type and S4 Fig). Anterior expansion of *Ptc1* was detected in E11.5 mutant limb buds (Fig 5G and 5H versus 5E and 5F in wild type and S4 Fig). In brief, our data revealed up-regulation of Shh signaling in limb buds lacking Sufu. It has been known that during limb bud patterning *Shh* maintains *Fgf* expression in the posterior AER through positively regulating expression of *Gremlin* in the posterior mesenchyme to counteract the negative regulation of BMP [2, 3]. The expression of *Gremlin* was anteriorly expanded in both the fore- and hind limb buds in *Sufu* ^*Dermo1Cre*^ by E10.5 (Fig 5K and 5O), compared to in wild type littermates (Fig 5I and 5M). In the E11.5 mutant limb bud, ectopically expressed *Gremlin* RNA was detected in the distal mesenchyme along the anterior and posterior axis (Fig 5L and 5P versus 5J and 5N in wild type). These data thus suggest that the Shh/Fgf signaling loop is ectopically activated in the anterior limb bud in mutants lacking Sufu, which indicates that Sufu is integrated in the Shh pathway to maintain the posterior-anterior patterning. The ectopically activated posterior markers *Shh*, *Gli1*, *Ptc1*, and *Gremlin* in the anterior limb bud, together with the anterior expanded *Fgf4* and *Fgf8* within the AER account for the development of the polydactylous digit pattern in *Sufu* ^*Dermo1Cre*^.

### Sufu deficiency results in the down-regulation of repressor Gli2/Gli3

**Fig 6:**
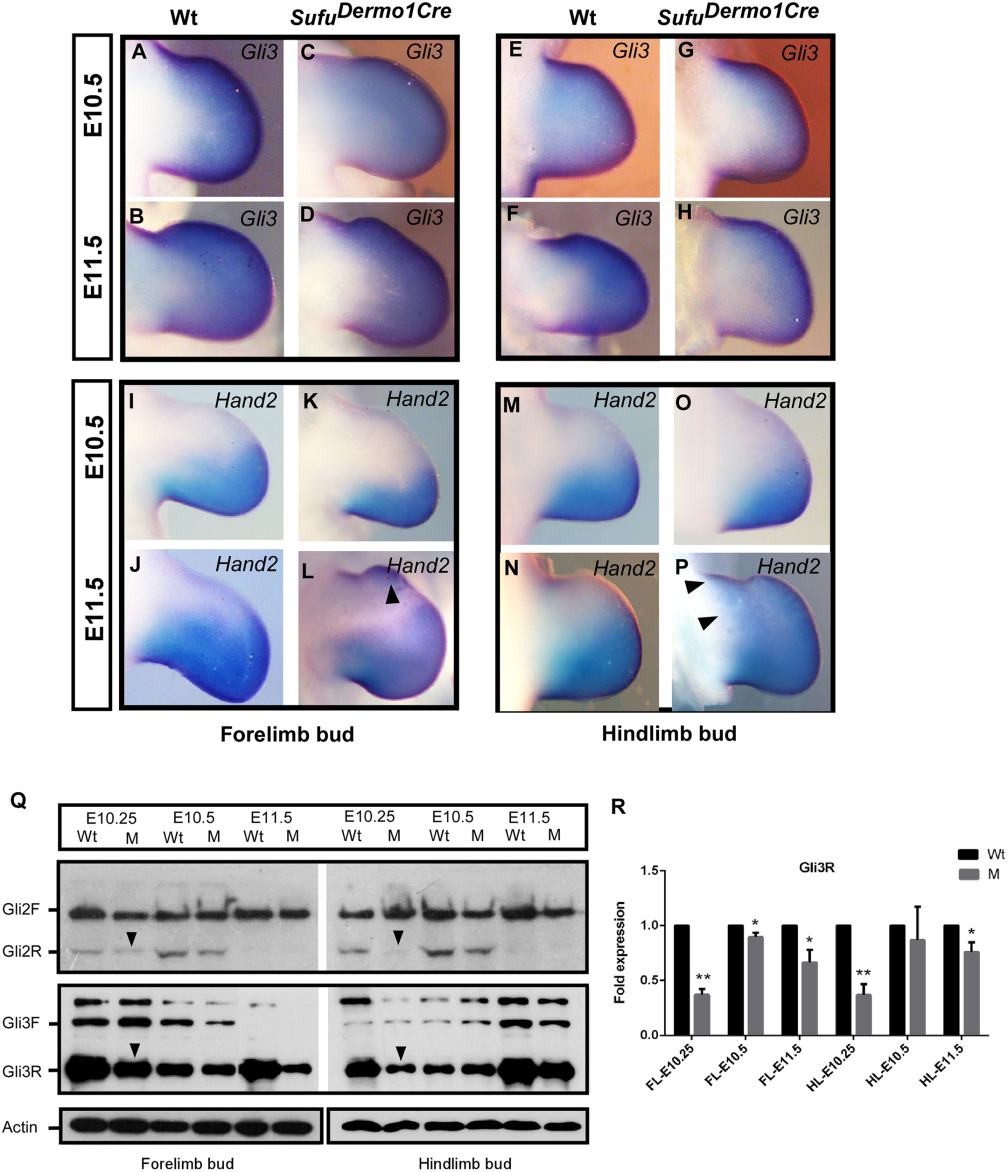
Sufu deletion cause alteration of Gli2R, Gli3R and *Hand2* expression. A-P: Whole-mount in situ hybridization showing the expression of *Gli3* and *Hand2*. Expression of *Gli3* in limb buds of the *Sufu* mutant (C, D, G, H) was comparable to the wild type (A, B, E, F). Anterior expansion of *Hand2* transcript was exhibited at E11.5 (L, P and arrowheads), compared to the wild type (J, N). Note the global distribution of *Hand2* transcripts in the entire hind limb bud (P versus L for forelimb bud). Q: Western blots show the decreased Gli2R protein (arrowhead) and Gli3R protein (arrowhead) in limb bud lysates at E10.25. R: Semi-quantitative analysis of the expression of Gli3R in Sufu mutant (M) limb buds compared with wild type (Wt). Student’s *t* test of significance with *P<0.05, **P<0.01.

Our in situ hybridization showed that the level of *Gli3* transcripts was not obviously changed in the *Sufu* ^*Dermo1Cre*^ limb bud (Fig 6A–6H), consistent with a previous study [23] in *Prx1-Cre*;*Sufu* ^*f/-*^ mutants where *Sufu* was also ablated in the mesenchyme of the early limb bud [23], suggesting that limb bud patterning defects might be due to the altered Gli3 in protein level. To examine the impact of Sufu deficiency on Gli3 forms in the early limb bud, we analyzed the protein lysates extracted from limb buds at E10.25, E10.5, and E11.5 by Western blot. Our results (n≥3) showed that there was a clear decrease in Gli3R level in the *Sufu* ^*Dermo1Cre*^ mutant compared to the wild type at E10.25 and E11.5 (Fig 6Q and S5 Fig), while there were no obvious variations of Gli3F compared to wild type control (Fig 6Q). Semi-quantitative analysis confirmed the reduction of Gli3R in the mutant at E10.25, E11.5, as well as E10.5 (Fig 6R). Given Sufu is a negative modulator promoting Gli3R formation in the absence of Shh [23], the anterior reduction of Gli3 repressor (Fig 6Q and 6R) in the *Sufu* mutant limb bud at E10.25 could be a consequence of Sufu loss of function. To further distinguish the Sufu and Shh regulation of Gli3 in the anterior and posterior regions, the forelimb buds at E10.25 and E10.5, when *Shh* was constrained in the posterior region, were divided into two halves along the central axis. Western blot analysis revealed that the Gli3R was primarily located in the anterior forelimb bud (S5 Fig). However, the decrease of Gli3R was more evident in the posterior region, likely related to different levels of Sufu deletion (S5 Fig and Fig 1). Nonetheless, our data confirmed that Sufu modulates Gli3R in early limb bud patterning, consistent with the recent report that showed failed establishment of A-P limb polarity with ectopic expression of posterior markers in the anterior of limb bud and down-regulation of anterior molecular markers in limb buds lacking Sufu [24].

While anterior Gli3R acts primarily in the establishment of limb A-P patterning [11, 12], Gli2Fcan compensate for Gli3F in posterior limb patterning [18]. To examine whether Sufu modulates Gli2R and Gli2F, we also analyzed the Gli2 protein by Western blot. Similar to Gli3R, the level of Gli2 (Gli2R) was reduced in the E10.25 *Sufu* ^*Dermo1Cre*^ limb buds (Fig 6Q). However, the Sufu deletion did not impact the level of full length Gli2 (Gli2F) (Fig 6Q). These data suggest that Sufu modulates the balance between the repressor and full-length forms of both Gli3 and Gli2 in the early limb bud, consistent with previous literature [18, 23].

To further examine whether loss of Sufu affects the establishment of A-P limb bud polarity through altering the antagonistic action of Gli3R and Hand2 in *Sufu* ^*Dermo1Cre*^, we examined the expression level of posterior marker *Hand2*. Coincident with the reduced *Gli3* in the anterior mesenchyme, the expression of *Hand2* was ectopically activated in the anterior forelimb bud at E11.5 of *Sufu* ^*Dermo1Cre*^ (Fig 6I–6L), while the *Hand2* transcripts are localized throughout the hind limb bud (Fig 6M–6P). Taken together, these data suggest that the establishment of A-P limb pattern is abrogated in the mutant limb bud lacking Sufu [20, 23]. To confirm this possibility, we examined the expression of *Pax9*, an anterior marker downstream of *Gli3* in the development of the anterior autopod [40, 41]. In situ hybridization exhibited that the *Pax9* expression in anterior limb buds was diminished in *Sufu* ^*Dermo1Cre*^ limb buds at E11.5 (S6 Fig), suggesting the altered anterior fate of the *Sufu* mutant limb bud.

In summary, while the essential role of Sufu in mouse development has been previously demonstrated [30], the in vivo genetic function of Sufu in regulation of limb bud patterning, which occurs after E9.5 in mouse embryogenesis when Sufu null-mutants die [30], has been unknown. By generating the mesenchyme-specific deletion of Sufu in limb buds, we present evidence that Sufu is directly involved in the determination of digit number and digit identity. Our data indicate that Sufu regulates digit patterning through the modulation of the *Shh/Gli3* pathway in the early limb bud, as exhibited by the digit phenotype similar among various Gli3 mutants [16, 18, 23]. Moreover, we also demonstrate that Sufu functions as a genetic modulator of both Gli2R and Gli3R levels in early limb bud patterning, consistent with the notion of genetic quantities of Gli2 and Gli3 as activators and repressors determine the regulation of the skeletal morphogenesis, digit number and digit identity [16, 18, 23]. We found that the repressor forms of Gli2 and 3 were significantly decreased in *Sufu* mutant limb buds at the early patterning stage, while the full length forms remained similar between the wild type and mutant, providing *in vivo* evidence that Sufu is a genetic regulator of Gli2 and Gli3 isoforms critical for limb bud patterning. In addition, our data that ectopic expression of posterior markers in the anterior limb bud lacking Sufu suggest that Sufu is a negative regulator of Shh signaling in early limb bud patterning [30]. It has been shown previously that the counteraction between Shh signaling and Gli3 determines the patterning along the anterior-posterior axis of the limb bud [16,17,21,24,40]. However, pre-patterning of the posterior specificity of the early limb bud prior to the activation of Shh signaling is established by anterior Gli3 restriction of posterior *Hand2* expression [20]. In the absence of Shh signaling, Gli3R acts to induce the AER and the ZPA, which are subsequently maintained by activated Shh signaling that antagonizes the formation of GliR, as described in detail previously [1, 11, 12, 16, 17, 23]. Unbalanced production of either Gli3R or Gli3F exhibited polysyndactyly and loss of digit identity, while the limb bud expressing both forms results in the development of a limb indistinguishable from the wild type [16, 17, 42]. On the other hand, premature activation of Shh signaling inhibited the formation of Gli3R [23], causing ectopic *Hand2* expression in the anterior limb bud [23] and thus the failure to establish the anterior and posterior limb identities. Once established in the posterior limb bud, Shh signaling is the major coordinator for Gli3R and Gli3F balance. Loss of Sufu in the early limb bud prior to the activation of Shh signaling reduced the levels of Gli3R, but did not significantly affect the level of Gli3F, suggesting that Sufu is indispensable for the formation of identity and the regulation of digit number via Gli3R. Moreover, our Gli2R data indicate that Sufu modulate Gli2R in digit patterning, supporting the importance of Gli2, together with Gli3, in regulation of the limb anterior-posterior polarity [18].

## Supporting Information

S1 Fig Gene targeting procedure for conditional *Sufu* knockout mouse. A: In the targeting construct, exon 7 of *Sufu* gene was flanked by two *LoxP* sites. B: Homologous recombination was genotyped by PCR using primer pairs designed in (A). C: Phenotype of *Sufu* ^*EIIaCre*^ at E9.5 with cephalic and neural tube defects.
(TIF)

S2 Fig Expression pattern of *Sufu* in the early developmental stages of limb buds. A-F: Whole-mount in situ hybridization shows expression of *Sufu*. Transcript is distributed in both the forelimb bud (A, C, and E) and the hind limb bud (D, F). B and B’ are horizontal sections of D and F, respectively.
(TIF)

S3 Fig *Shh* expression in limb bud of *Sufu* ^Dermo1Cre^. A-J: Whole-mount in situ hybridization showing the *Shh* expression pattern in the Sufu mutant (D, E, I, J) and the wild type (A, B, G, H).
(TIF)

S4 Fig Expression of *Gli1* and *Ptc1* in the forelimb bud. A-H: Whole-mount in situ hybridization showing the anterior expansion of the *Gli1* transcript (A–D) and the *Ptc1* transcript (E–H).
(TIF)

S5 Fig Immunoblot analysis of the effect of Sufu deletion on the Gli3 forms. A: Western blot shows the Gli3 isoforms affected by Sufu deletion in the anterior and posterior of forelimb buds at E10.25 and E10.5. B:Semi-quantitative analysis of the expression of Gli3R in Sufu mutant (M) limb buds compared with wild type (Wt). FLA: anterior of forelimb bud; FLP: posterior of forelimb bud. Student’s *t* test of significance with *P<0.05, **P<0.01.
(TIF)

S6 Fig Expression of anterior marker gene. A-D: Whole-mount in situ hybridization showing the diminished *Pax9* expression in the E11.5 mutant limb buds lacking Sufu (C, D versus A, B for wild type).
(TIF)
