# Supplementary figures and images for: Suppressor of Fused Is Required for Determining Digit Number and Identity via Gli3/Fgfs/Gremlin
Source: PLoS One. 2015 May 22;10(5):e0128006. doi: 10.1371/journal.pone.0128006 (PMC4441507; doi:10.1371/journal.pone.0128006)

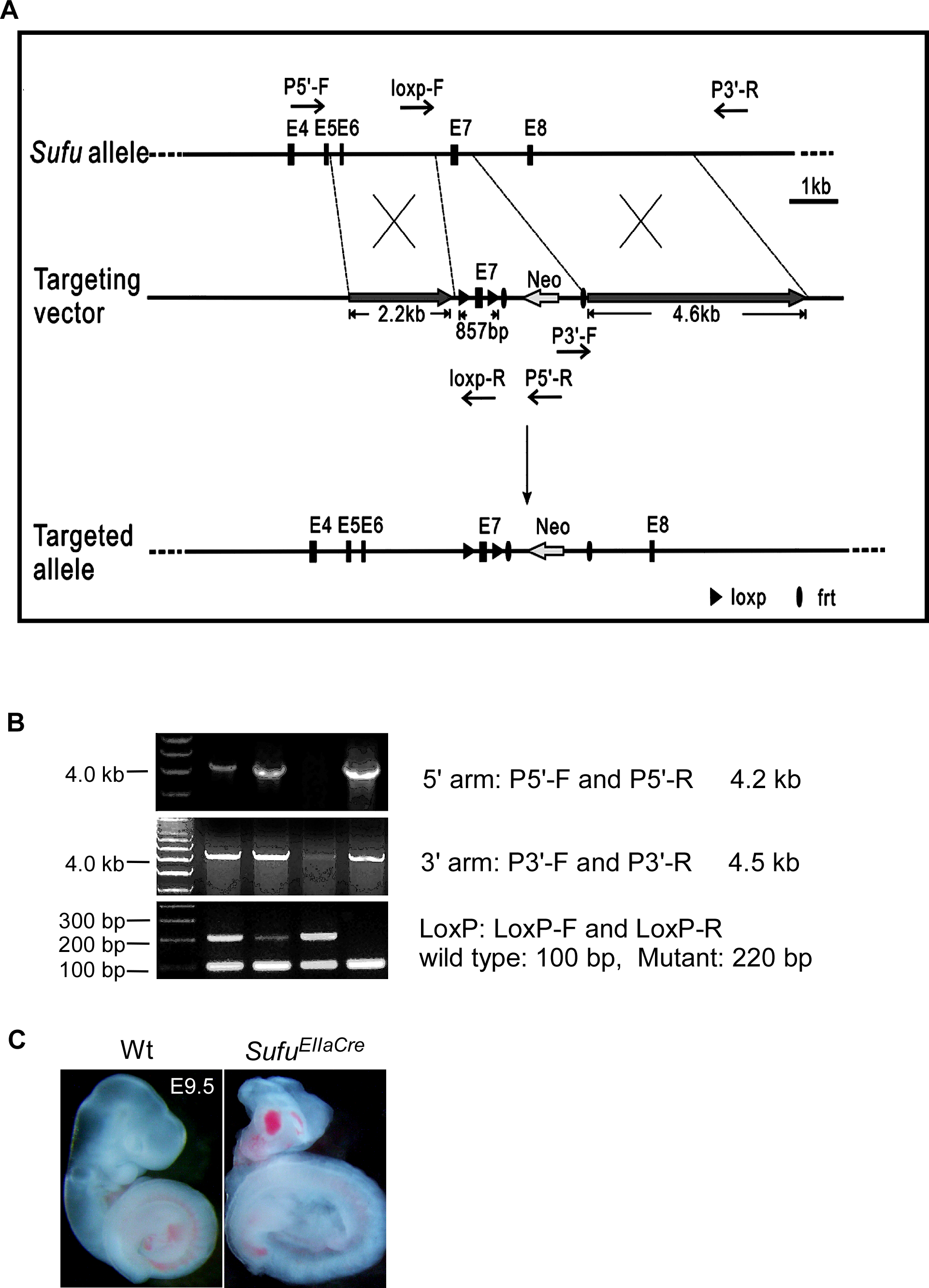

Supplement: S1 Fig — A: In the targeting construct, exon 7 of Sufu gene was flanked by two LoxP sites. B: Homologous recombination was genotyped by PCR using primer pairs designed in (A). C: Phenotype of Sufu EIIaCre at E9.5 with cephalic and neural tube defects. (TIF) [file pone.0128006.s001.tif]

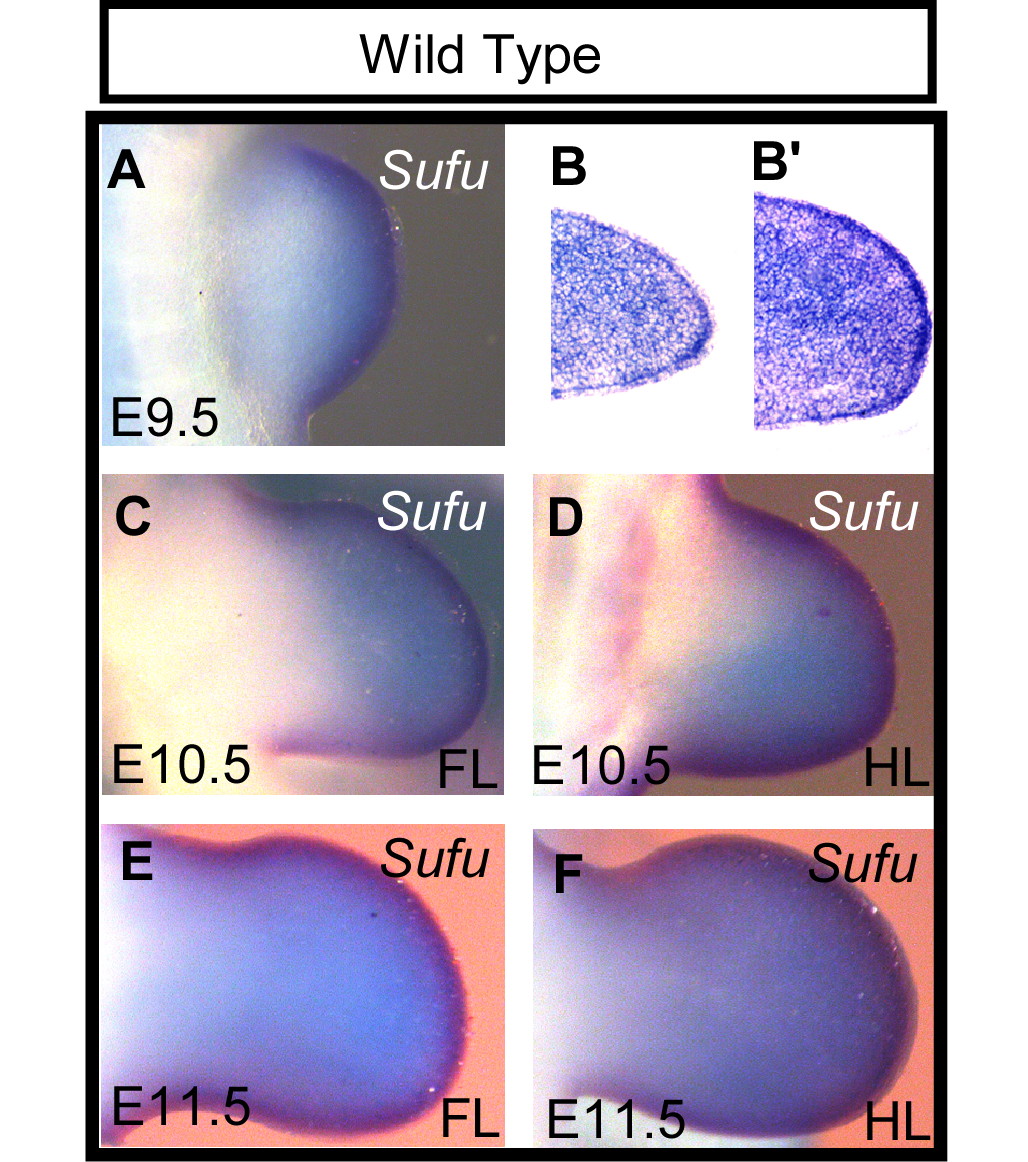

Supplement: S2 Fig — A-F: Whole-mount in situ hybridization shows expression of Sufu. Transcript is distributed in both the forelimb bud (A, C, and E) and the hind limb bud (D, F). B and B’ are horizontal sections of D and F, respectively. (TIF) [file pone.0128006.s002.tif]

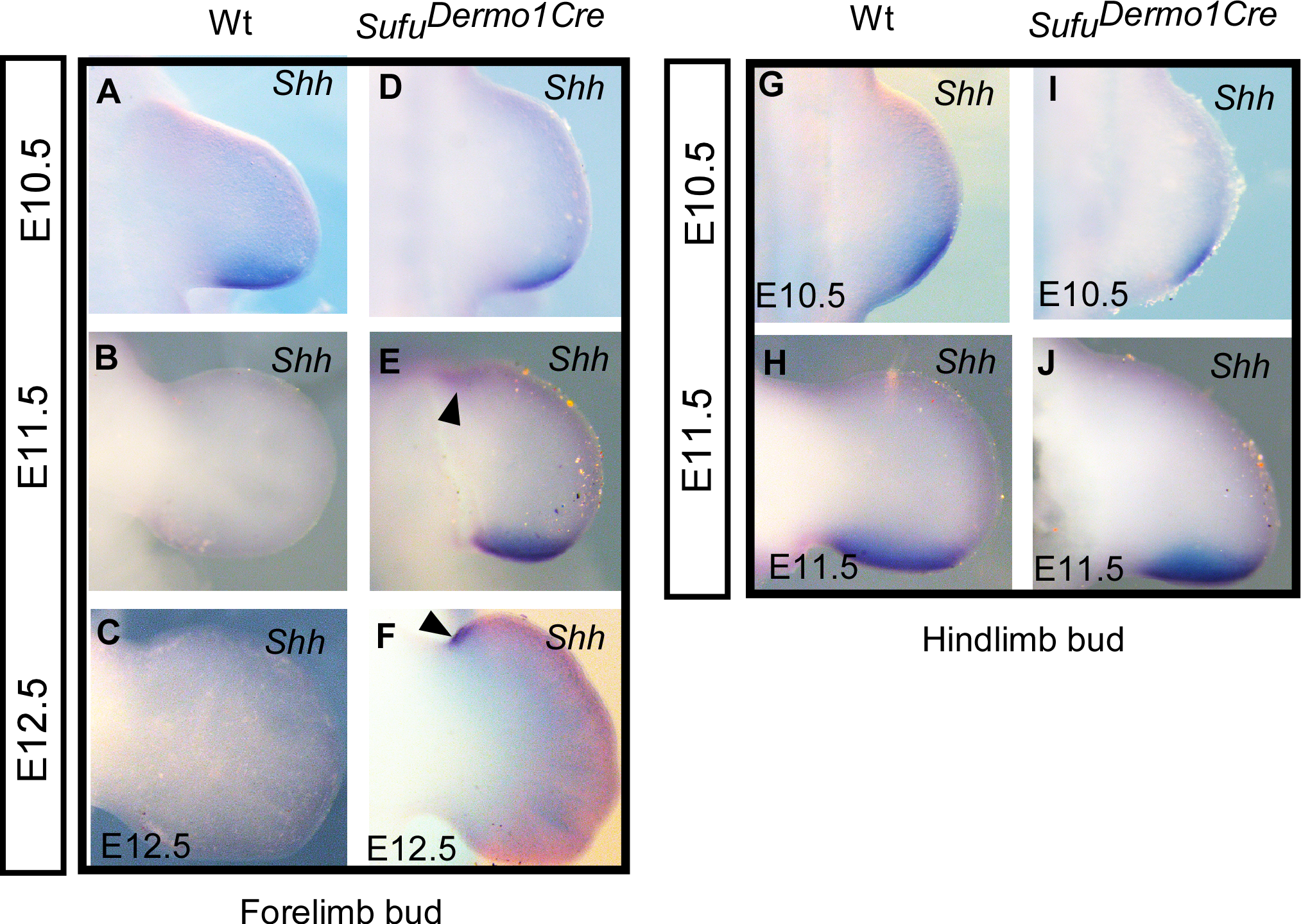

Supplement: S3 Fig — A-J: Whole-mount in situ hybridization showing the Shh expression pattern in the Sufu mutant (D, E, I, J) and the wild type (A, B, G, H). (TIF) [file pone.0128006.s003.tif]

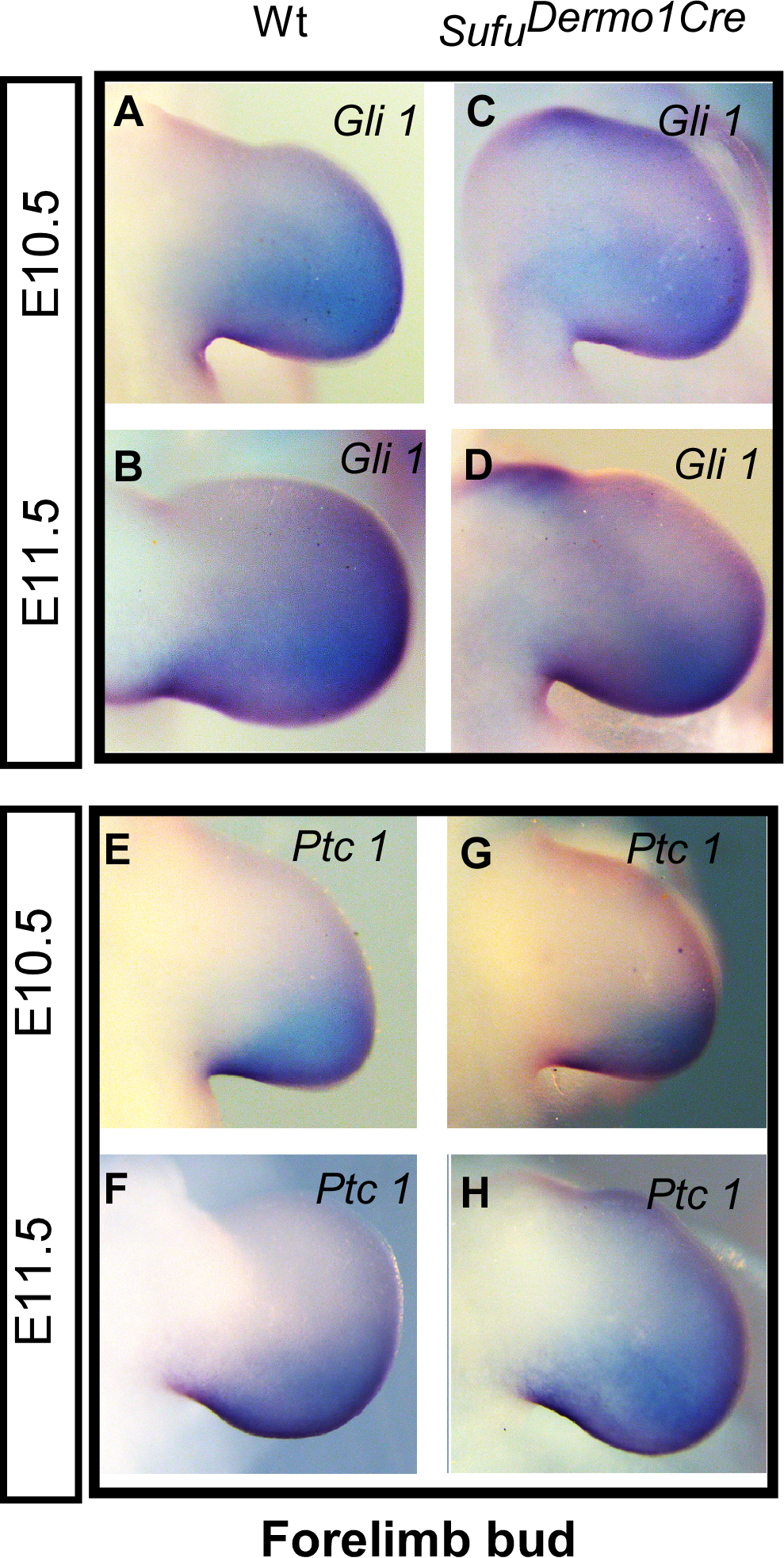

Supplement: S4 Fig — A-H: Whole-mount in situ hybridization showing the anterior expansion of the Gli1 transcript (A–D) and the Ptc1 transcript (E–H). (TIF) [file pone.0128006.s004.tif]

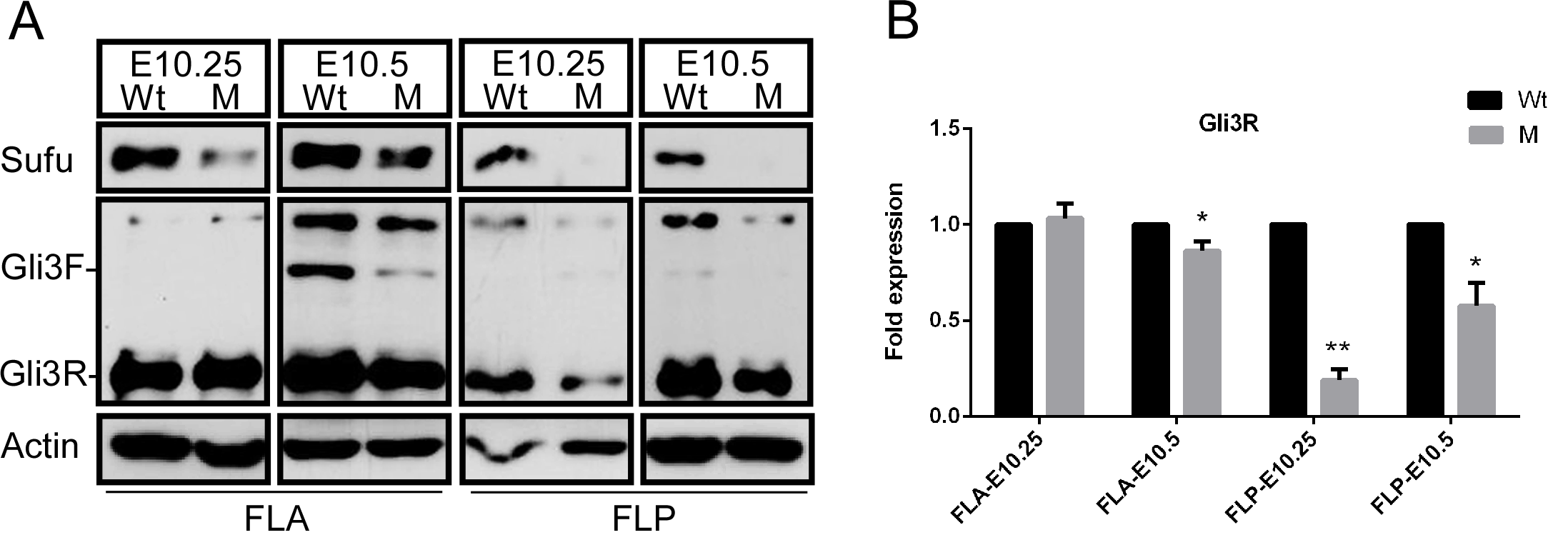

Supplement: S5 Fig — A: Western blot shows the Gli3 isoforms affected by Sufu deletion in the anterior and posterior of forelimb buds at E10.25 and E10.5. B:Semi-quantitative analysis of the expression of Gli3R in Sufu mutant (M) limb buds compared with wild type (Wt). FLA: anterior of forelimb bud; FLP: posterior of forelimb bud. Student’s t test of significance with *P<0.05, **P<0.01. (TIF) [file pone.0128006.s005.tif]

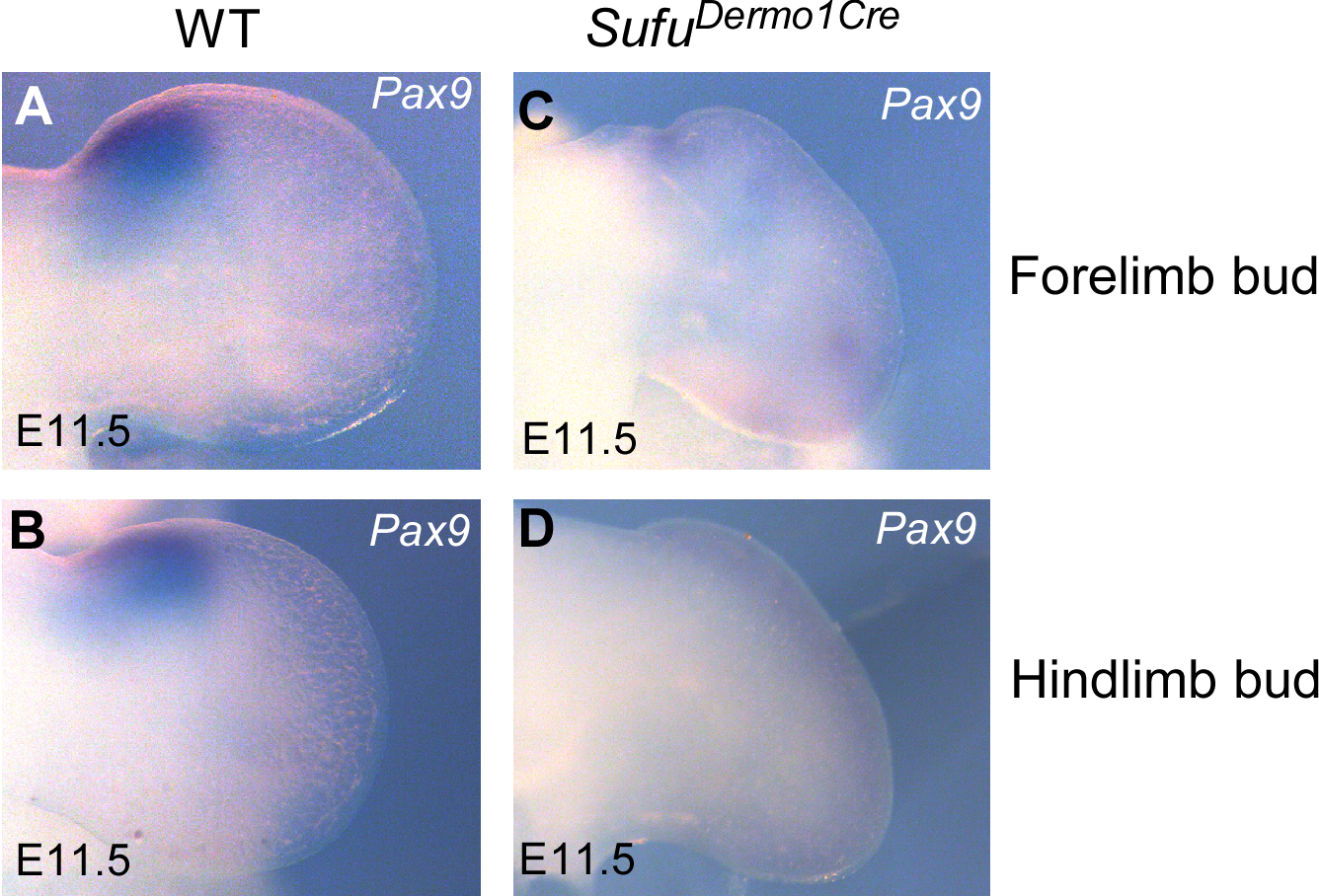

Supplement: S6 Fig — A-D: Whole-mount in situ hybridization showing the diminished Pax9 expression in the E11.5 mutant limb buds lacking Sufu (C, D versus A, B for wild type). (TIF) [file pone.0128006.s006.tif]
